# Effect of hypoxia on GLP-1 secretion – an in vitro study using enteroendocrine STC-1 -cells as a model

**DOI:** 10.1007/s00424-024-02996-z

**Published:** 2024-07-29

**Authors:** Ravikant Sharma, Ghulam Shere Raza, Nalini Sodum, Jaroslaw Walkowiak, Karl-Heinz Herzig

**Affiliations:** 1 Research Unit of Biomedicine and Internal Medicine, Biocenter of Oulu, Medical Research Center, University of Oulu, Aapistie 5, 90220 Oulu, Finland; 2 Department of Gastroenterology and Metabolism, Poznan University of Medical Sciences, 60572 Poznań, Poland

**Keywords:** Hypoxia, GLP-1, Enteroendocrine cells, Fatty acid, GPR40 and GPR120

## Abstract

Glucagon-like peptide (GLP)-1 is a hormone released by enteroendocrine L-cells after food ingestion. L-cells express various receptors for nutrient sensing including G protein-coupled receptors (GPRs). Intestinal epithelial cells near the lumen have a lower O_2_ tension than at the base of the crypts, which leads to hypoxia in L-cells. We hypothesized that hypoxia affects nutrient-stimulated GLP-1 secretion from the enteroendocrine cell line STC-1, the most commonly used model. In this study, we investigated the effect of hypoxia (1% O_2_) on alpha-linolenic acid (αLA) stimulated GLP-1 secretion and their receptor expressions. STC-1 cells were incubated for 12 h under hypoxia (1% O_2_) and treated with αLA to stimulate GLP-1 secretion. 12 h of hypoxia did not change basal GLP-1 secretion, but significantly reduced nutrient (αLA) stimulated GLP-1 secretion. In normoxia, αLA (12.5 μM) significantly stimulated (~ 5 times) GLP-1 secretion compared to control, but under hypoxia, GLP-1 secretion was reduced by 45% compared to normoxia. αLA upregulated GPR120, also termed free fatty acid receptor 4 (FFAR4), expressions under normoxia as well as hypoxia. Hypoxia downregulated GPR120 and GPR40 expression by 50% and 60%, respectively, compared to normoxia. These findings demonstrate that hypoxia does not affect the basal GLP-1 secretion but decreases nutrient-stimulated GLP-1 secretion. The decrease in nutrient-stimulated GLP-1 secretion was due to decreased GPR120 and GPR40 receptors expression. Changes in the gut environment and inflammation might contribute to the hypoxia of the epithelial and L-cells.

**Graphical Abstract:**

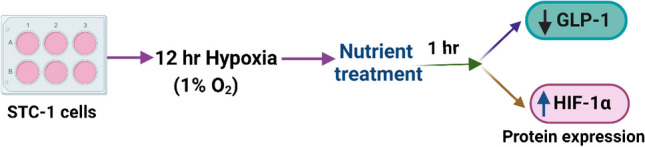

## Introduction

Glucagon-like peptide-1 (GLP-1) is an amino acid peptide hormone in different molecular forms (GLP-1 (1–37; 7–37; 7–36), released by enteroendocrine L-cells in response to nutrient load [13]. GLP-1 is an incretin that regulates blood glucose levels by insulin release from the pancreatic β-cells, food intake, and gut motility [27]. In human plasma, active forms are GLP-1 (7–36) amide and (7–37) amide, which are rapidly degraded by dipeptidyl peptidase 4 (DPP4) into GLP-1 (9–36) amide and (9–37) amide. Due to its quick inactivation, GLP-1 has a short half-life of less than 2 min and only 10–15% of the released GLP-1 enters the systemic circulation [2]. In humans, L-cells are localized in the proximal small intestine and progressively increase in density towards the distal part of the intestine and colon [27]. GLP-1 plasma concentrations are low (5–15 pmol/L) in the fasted state and increase 2- to 4 fold postprandially by nutrients such as carbohydrates, peptides, and lipids [7]. The apical surface of L-cells allows direct interaction with nutrients [8]. Sodum et al. recently reported that nutrient combinations such as amino acid and fatty acid potentiate GLP-1 secretion compared to individual nutrients in the L-cells [48]. L-cells express several receptors for nutrient sensing including G protein-coupled receptors (GPRs) [44]. Long chain fatty acids (LCFAs) including oleic acid, linolenic acid, alpha-linolenic acid (αLA), eicosadienoic acid, and arachidonic acid activate GPR120, GPR40, GPR119, inducing GLP-1 secretion [12]. Hirasawa et al. reported GPR120 but not GPR40, mediates FFA-induced Ca^2+^ response and GLP-1 secretion from STC-1 cells [26].

Oxygen (O_2)_ is a key factor affecting GLP-1 synthesis and release [55]. The epithelial cells near the lumen have a much lower O_2_ tension than at the base of the crypts with a steep oxygen gradient, which is critical for intestinal homeostasis [46]. The O_2_ tension in the villus falls significantly from 14–17 mmHg in the interdigestive phase to 4–7 mmHg postprandial [17]. Furthermore, O_2_ levels drop from 10–7.5% in the stomach to 1.5—0.5% at the colon-rectal junction [58]. These anatomical characteristics provide a sharp oxygen gradient across the tissue, making intestinal cells hypoxic even under physiological conditions, known as 'physiological hypoxia'. As a consequence, the colonic microbiome is primarily composed of anaerobic bacteria, predominantly Firmicutes, Bacteroidetes, Actinobacteria, Proteobacteria, and Verrucomicrobia [41]. Anaerobes further contribute to the intestine's hypoxic environment by utilizing part of the O_2_ from the bloodstream [8].

Hypoxia induces the stabilization of the transcription factors hypoxia-inducible factor-1α (HIF-1α) and HIF-2α which is influenced by O_2_-regulated proteins such as prolyl-hydroxylases (PHDs) and factor-inhibiting HIF (FIH) [21]. The HIF-α subunit has three isoforms in vertebrate species: HIF-1α, HIF-2α, and HIF-3α. These proteins enter the nucleus and form complexes with the constitutively produced β subunit, regulating gene expression of several fundamental cellular processes such as metabolism and autophagy.

There are conflicting results on the effect of hypoxia on GLP-1 secretions: Moderate hypoxia (15.0% O_2_) for 7 h in healthy human subjects did not change plasma GLP-1 levels [39]. In addition, rats exposed to hypobaric hypoxia (simulated altitude of 7620 m) for 6-168 h decreased plasma CCK and GLP-1 [15]. In contrast, 64 cycles of intermittent hypoxia for 5 min (1% O_2_) and 10 min normoxia (21% O_2_) increased mRNA expression of *PYY* and GLP-1 in STC-1 cells [45].

We hypothesized that hypoxia might affect nutrient-stimulated GLP-1 levels from the mouse enteroendocrine cell line STC-1 [29]. Our study investigated the effect of hypoxia (1% O_2_) on nutrient-stimulated (αLA) GLP-1 secretion.

## Materials and methods

### Materials

Dulbecco’s modified eagle medium (DMEM, Cat. No. P04-03500 PAN Biotech, Aidenbach Germany), α-Linolenic Acid (Cat. No. L2376), DPP4 inhibitor (Cat. No. DPP4-010), and active GLP-1 ELISA kit (Cat. No. EGLP-35 K) were purchased from Merck (Darmstadt, Germany), Horse serum (Cat. No. 16050–122, ThermoFisher, Waltham, MA), fetal bovine serum (Cat. No. 10270106, ThermoFisher), L-glutamine Gibco (Cat. No. 25030–024, ThermoFisher), penicillin–streptomycin Gibco (Cat. No. 15140–122, ThermoFisher), protease inhibitor cocktail (Cat. No. 10516495, Sigma-Aldrich, St. Louis, MO), T-75 and 6 wells plates (Sarstedt AG & Co. KG, Nümbrecht, Germany), HIF-1α rabbit monoclonal antibody (Cat. No. D1S7W 36169, Cell Signaling Technology, Danvers, MA), GPR120 polyclonal antibody (Cat. No. PA5-50,973, ThermoFisher Scientific, Rockford, IL), GPR40 polyclonal antibody (Cat. No. PA5-67,931, ThermoFisher Scientific, Rockford, IL) GAPDH rabbit monoclonal antibody (Cat No. 2118 14C10, Cell Signaling Technology, Danvers, MA), Goat anti-rabbit IgG antibody (Cat. No. ab6721, abcam, Cambridge, United Kingdom), PVDF membrane (Cat No. 24937–79-9 Merck Millipore, Burlington, MA), Super Signal TM West Femto Substrate (Cat. No. 34095, Thermo Fisher Scientific, Rockford, IL). All reagents used were of analytical grades.

### STC-1 cell culture

The STC-1 cell line was cultured in T75 flasks in DMEM supplemented with 15% horse serum, 2.5% fetal bovine serum (FBS), 1% L-glutamine, and penicillin–streptomycin as previously described [40]. Cells were maintained in the above media at 37˚C, 5% CO_2_ until 85–90% confluency. Passage numbers 14–21 were used for the experiment. The mouse STC-1 cell line is the most commonly used GLP-1 secreting cell line and serves as a model cell line in the investigations of GLP-1 [26, 29, 40, 48]. In addition, other cell lines like mouse GLUTag cells secrete significantly less GLP-1 [29]. Human cell lines secreting GLP-1 are the colon cancer cell line Caco-2 and the NCI-H716 cell line from a 33-yr-old Caucasian male with poorly differentiated adenocarcinoma of the colon [42, 50]. Cell line models of L-cells differ from each other and from the natural L-cells [36]. However, there is abundant GLP-1 in the distal intestine in different species like mice, rats, pigs and humans responding to nutrient stimulation [30].

### GLP-1 secretion in STC-1 cells under normoxia and hypoxia

**Fig. 1:**
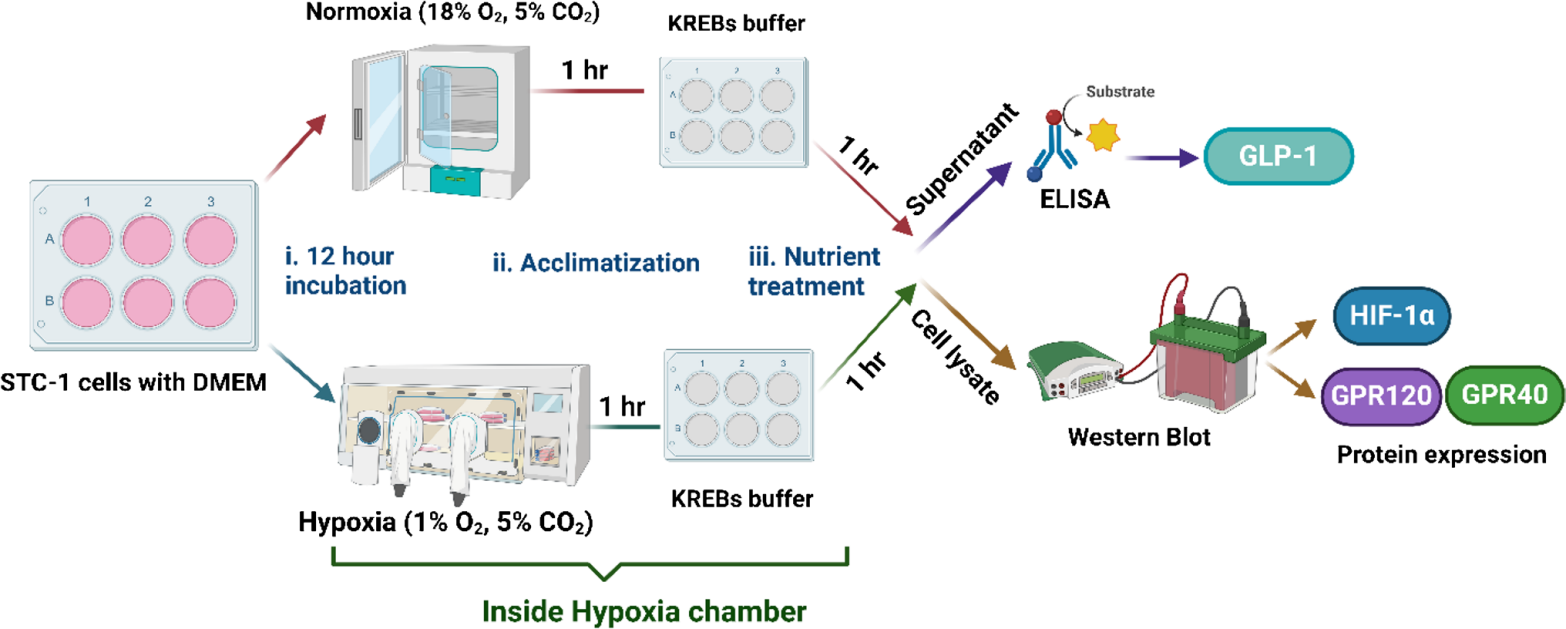
Study design. STC-1 cells were incubated with DMEM under normoxia and hypoxia (1% O_2_, 5% CO_2_) for 12 h and treated with αLA (12.5 μM) for an additional 1 h. GLP-1 release were analyzed in the supernatant and cell lysates were used for protein expression of HIF-1α, GPR120 and GPR40

STC-1 cells were seeded at 2.0 × 10^6^ cells/well with 2 ml media in 6-well cell culture plates. Cells were counted by an automated cell counter (LUNA-II™, Logos Biosystems, Inc., Villeneuve-d´Ascq, France). On the day of experiments, cells were washed and incubated with DMEM for 12 h in normoxia (18% O_2_, 5% CO_2_) and hypoxia (1% O_2_, 5% CO_2_) using the hypoxic chamber, Sci-Tive-N/Ruskinn, Baker, Sanford, ME. After 12 h of incubation, the cells were washed twice with Krebs–Ringer Bicarbonate Buffer (KREBS; 118 mM NaCl, 4.7 mM KCl, 25 mM NaHCO_3_, 1.25 mM CaCl_2_, 1.2 mM MgSO_4_, and 1.2 mM KH_2_PO_4_) pH 7.4. Hypoxia was maintained throughout the experiment by keeping the cells in a hypoxia chamber and all the treatments including washing were performed inside the chamber. Following washing cells were acclimatized with KREBS buffer for 1 h. After 1 h of acclimatization, the buffer was aspirated, and cells were treated in triplicates with different treatments along with DPP4 inhibitor in KREBS buffer for 1 h at 37^◦^C: αLA (12.5 μM) as shown in Fig. 1 [48]. KREBS buffer with ethanol (0.1%) and DPP4 inhibitor (0.25%) was used as a control. After 1 h of treatment, the supernatant was collected, centrifuged at 13,000 g for 15 min at 4^◦^C and stored at -70^◦^C. Cells were washed with ice-cold PBS for protein analysis. Active GLP-1 was measured by an active GLP-1 ELISA kit as per manufacturer instructions [29] (*n* = number of individual experiments performed in triplicates).

## Immunoblotting for protein analysis

Cells were scrapped from a 6-well plate using a cell scrapper and ice-cold RIPA lysis buffer (50 mM Tris, 150 mM NaCl, 0.1% Triton X-100, 0.5% sodium deoxycholate, 0.1% SDS), containing protease inhibitor cocktail (Sigma, St. Luis, MO). The suspension was passed using a 27G needle and centrifuged at 13,000 g for 20 min at 4˚C. Total protein concentrations were determined by the Bradford reagent (Bio-Rad Laboratories Inc., Hercules, CA). Equal amounts of protein (12.5 μg) were separated by 8% SDS-PAGE gel and transferred to a PVDF membrane. The membrane was blocked using 5% skim milk in Tris-buffered saline containing 0.1% Tween 20 (TBST) for 1 h and incubated with primary HIF-1α monoclonal antibody (1:1000), GPR120 polyclonal antibody (1:1000), GPR40 polyclonal antibody (1:1000) and loading control protein GAPDH (1:1000) overnight at 4˚C. After overnight incubation, the membrane was incubated with a secondary antibody (1:10,000 Goat anti-rabbit IgG horseradish peroxidase-conjugated anti-rabbit IgG) for 1 h at room temperature. Chemiluminescence for proteins was detected using SuperSignal™ West Femto Maximum Sensitivity Substrate according to the manufacturer´s instructions. Blots were visualized with an Odyssey Fc imaging system (LI-COR Biosciences, Ltd, Cambridge, UK).

### Statistical analysis

One-way analysis of variance (ANOVA) was used to analyze statistical significance between the groups using GraphPad Prism, version 7 (GraphPad Software, Inc., La Jolla, CA). Dunnett’s multiple comparison test was used to analyze the difference between the treatment groups. The values are represented as mean ± standard errors of the mean (SEM) and differences were considered statistically significant when *p* < 0.05.

## Results

### In vitro GLP-1 secretions in STC-1 cells

**Fig. 2:**
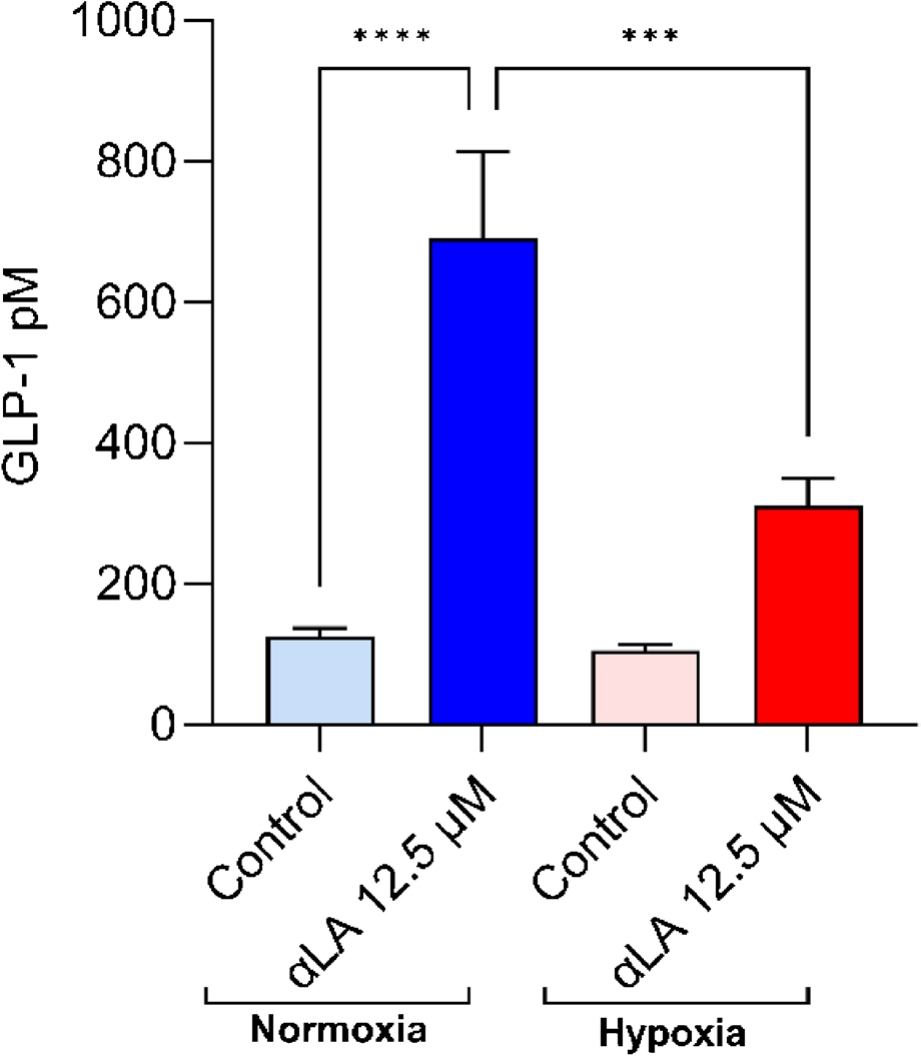
Active GLP-1 secretion under normoxia and hypoxia in STC-1 cells. 12 h hypoxia did not significantly affect basal GLP-1 secretion compared to normoxia control. αLA significantly stimulated GLP-1 secretion under normoxia (~ 7 folds), but no significant change under hypoxia compared to buffer (control). In addition, αLA-stimulated GLP1 secretion under hypoxia was significantly lower compared to normoxia αLA. *n* = 5 (*n* = number of individual experiments in triplicates). The value represents the mean ± standard error of the mean (SEM) and differences were considered statistically significant when ***p < 0.001 and ****p < 0.0001

12 h hypoxia did not significantly affect basal GLP-1 secretion, but nutrient (αLA) stimulated GLP-1 secretion was significantly reduced by 45% (Fig. 2). αLA significantly stimulated GLP-1 secretion under normoxia (~ 7 folds), but no significant change under hypoxia in STC-1 cells compared to buffer (control) (Fig. 2). In addition, αLA-stimulated GLP-1 secretion under hypoxia was significantly lower compared to normoxia αLA (Fig. 2). A total of 5 independent experiments were performed in triplicates.

### HIF-1α protein expression in STC-1 cells

**Fig. 3:**
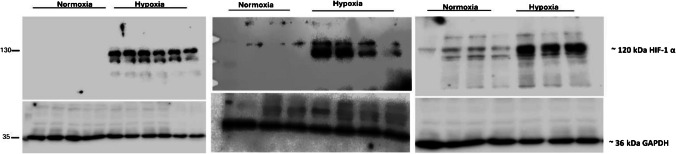
Representative western blots of HIF-1α in STC-1 cells. In hypoxic conditions, all the blots showed intense HIF-1α protein bands at ~ 120 kDa and only faint bands under normoxia. **A**) 12 wells of 2 experiments in triplicates. **B**) 4 wells from one experiment in triplicates plus one additional well from another experiment under hypoxia. **C**) 7 wells from one experiment in triplicates and one additional well under normoxia

All the blots clearly show the HIF-1α protein bands at ~ 120 kDa. 12 h hypoxia (1% O_2_) noticeably induced HIF-1α protein expressions in STC-1 cells (Fig. 3). Under normoxia, only faint HIF-1α protein bands were observed (Fig. 3). These blots clearly show that hypoxia was established in STC-1 cells as intense HIF-1α protein bands were visible.

## GPR120 and GPR40 protein expression

**Fig. 4:**
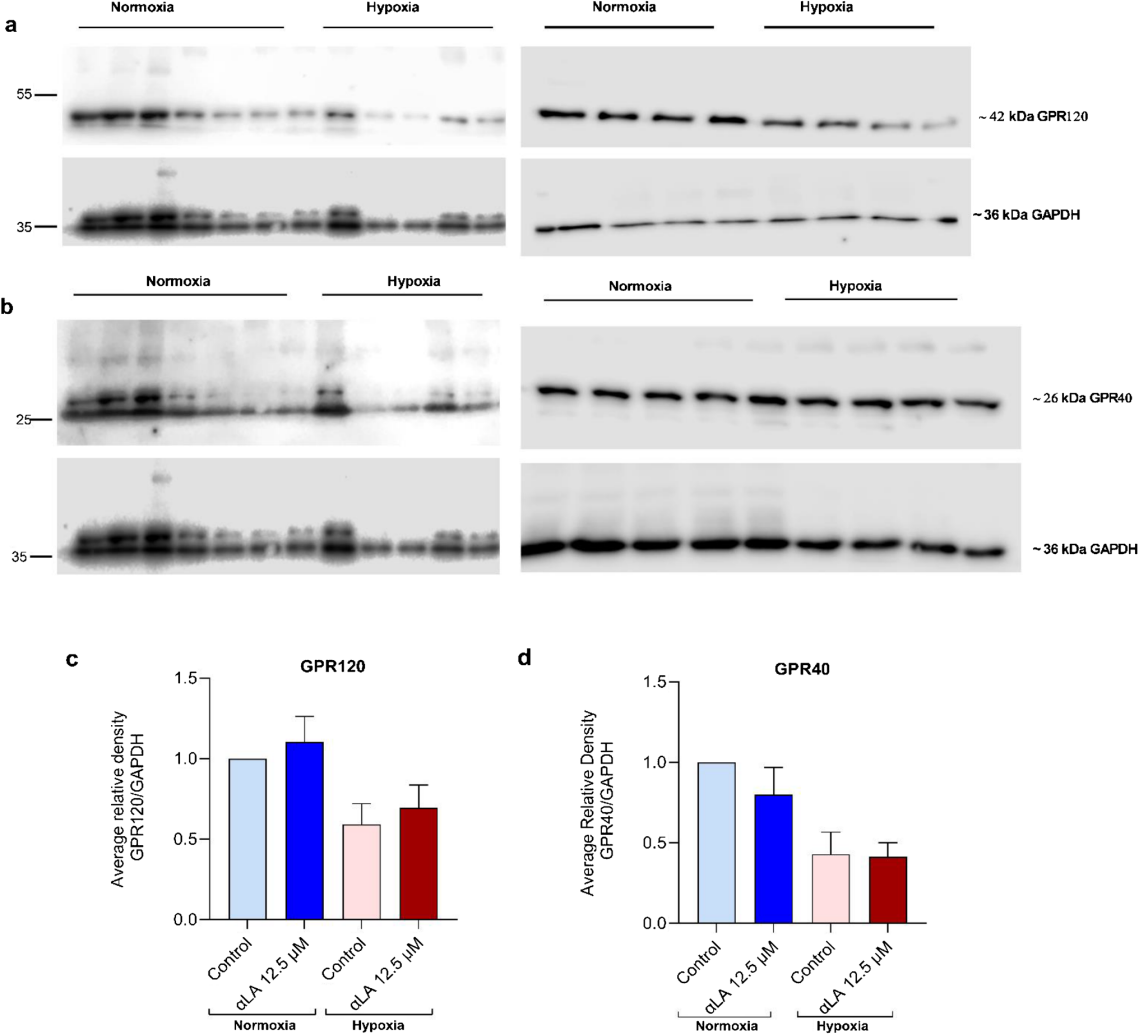
Protein expression for (**a**) GPR120 blots (**b**) GPR40 blots (**c**) Average Relative Density of GPR120 with GAPDH (**d**) Average Relative Density of GPR40 with GAPDH. Hypoxia downregulated GPR120 and GPR40 protein expressions in STC-1 cells. The value represents the mean ± standard error of the mean (SEM) and differences were considered statistically significant when *p* < 0.05 and *n* = 4

All blots showed the bands for GPR120 at ~ 43 kDa (Fig. 4a) and for GPR40 at ~ 26 kDa (Fig. 4b). Our results demonstrated that hypoxia downregulated GPR120 and GPR40 expressions by 50% and 60% respectively, compared to normoxia (Fig. 4c). αLA upregulated GPR120 expression (10%), while GPR40 was downregulated (20%) under normoxia (Fig. 4d). αLA did not significantly change either of the receptors on the protein expression levels under hypoxia (Fig. 4d).

## Discussion

Our study demonstrated that nutrient αLA significantly stimulated GLP-1 secretion from enteroendocrine STC-1 cells under normoxia but not under hypoxic conditions. However, no significant change in GLP-1 secretion was found under basal conditions between hypoxia and normoxia. αLA was used as a positive control to stimulate GLP-1 secretion in STC-1 cells as previously described [48]. Our results are consistent with previous findings under normoxia on GLP-1 secretion with fatty acids in STC-1 cells [29, 48]. Consistent with our findings on hypoxia, Kondrashina et al. showed that casein-stimulated GLP-1 secretion significantly decreased under hypoxia (7.5% O_2_) in STC-1 cells [34]. In addition, hypoxia (1% O_2_) decreased forskolin-stimulated GLP-1 secretion from enteroendocrine GLUTag cells [32]. The author suggested that the decrease in GLP-1 secretion could be due to reduced GLP-1 synthesis [32]. 6-168 h of hypoxia (simulated altitude of 7620 m) in rats decreased plasma CCK and GLP-1 [15]. In contrast, it has been shown that 64 cycles of intermittent hypoxia for 5 min (1% O_2_) and 10 min normoxia (21% O_2_) increased mRNA expression of PYY and GLP-1 in STC-1 cells [45]. Chronic hypoxia (14.4—14.7% O_2_) for four weeks, lowered body weight, and improved glucose, and lipid metabolism in HFD-induced obese mice [54]. In humans, normobaric hypoxia (fraction of inspired oxygen: FiO_2_:0.12 ~ 5000 m) for 10 h reduced appetite and hunger compared to normobaric normoxia (FiO_2_:0.21), but gut peptides were unfortunately not measured [4]. 17 h hypoxic exposure (12.5% inspired O_2_, simulating approximately ~ 4100 m) in humans increased plasma leptin and only a small increase in postprandial GLP-1 levels after 40 min [47]. In humans, reduced energy intake, plasma acylated ghrelin concentrations, and PYY concentrations were reported at a simulated altitude of 4000 m [56]. Matu et al. reported lower appetite and post-exercise acetylated ghrelin area under the curve at an altitude of 4300 m, but no change in GLP-1 secretions [37]. A reason might be that no DPP4 was added for the measurement of GLP-1.

Several peptides including GLP-1 are secreted from the intestinal tract, which has a distinct oxygen gradient. A vertical oxygen gradient has been found in the more distal colonic parts of the gastrointestinal (GI) tract. The epithelial cells lining of intestinal mucosa reside in a relatively low pO_2_ environment [10]. The pO_2_ of the colonic muscle wall is 42–71 mmHg (7–10%), vascularized submucosa 42 mmHg (6%), crypt-lumen interface 5–10 mmHg, ascending colon 11 mmHg (2%) and sigmoid colon 3 mmHg (0.4%) [25]. During low-oxygen conditions, cells adapt to hypoxic stress by increasing the HIFs expression, which regulates metabolic processes and energy metabolism [43]. Hence in our study, we incubated STC-1 cells for 12 h (1% O_2_, 5% CO_2_) to induce and stabilize HIF-1α. HIF-1α protein expressions clearly state that hypoxia was maintained throughout the experiment **(**Fig. 3**)**. We observed faint HIF-1α protein bands under normoxia, which has also been reported in other studies [18, 19, 49]. Acute infection and inflammation cause significant changes in tissue metabolism, resulting in severe tissue hypoxia [22]. A study on murine dendritic cells subjected to hypoxia (2% oxygen for 24 h) demonstrated an elevation in transcript levels of Toll-like receptors TLR2 and TLR6 [35]. In addition, hypoxic environment affects the intestinal microbiome. Following a high-fat diet (HFD) through meat consumption, both humans and animals showed a higher abundance of Firmicutes and a decrease in Bacteroidetes. Changes in the gut microbiota populations activate the Toll-like receptor (TLR) signaling pathway, resulting in increased intestinal permeability to endotoxins [12]. In mice colon, Bacteroidetes were reported about 3 times higher in abundance during hypoxia (simulated altitude of 5500 m for 24 h) [53]. In mice, the ratio of total aerobic to anaerobic bacteria changed from 1:2.79 to 1:7.34 under exposure to a high-altitude environment for 30 days and the total number of anaerobes increased about 105 times higher [1]. The *Bacteroidetes* abundance increased from days 1 to 14 in mice during environmental hypoxia with HIF-1ß deficiency in their bone marrow cells [23]. These disturbances in gut microbiota might cause a reduction in GLP-1 secretion. Colonocytes use butyrate produced by the bacteria as an energy substrate and maintain the anaerobic environment in the lumen [11]. In Caco-2 cells, butyrate increased O_2_ consumption and stabilized HIF-1α, leading to lower barrier permeability [31]. In human NCI-H716 cells, butyrate stimulated dose-dependently and biphasic GLP-1 secretion [57]. Yet, butyrate promotes epithelial barrier function by depleting oxygen levels near the epithelium and stabilizing HIF [3], stimulating mucin production [24]. The microbiome generates biofilms, which further contributes to the decreased oxygen gradient in the colon [5, 51]. Oxygen concentrations are high at the liquid surface and low in the deeper parts of the biofilm. In addition, the oxygen diffusion rate (about 60% of the rate observed in water) is lower in biofilms [33] which also contributes to the hypoxic environment in the colon.

Intestinal HIF affects GLP-1 secretion via the lipid sensor G-protein–coupled receptor enriched in L-cells. The enteroendocrine cells sense fatty acids (FAs) via GPR120 and GPR40 [52]. Thus, we investigated GPR120 and GPR40 protein expressions. We found that hypoxia (increased HIF-1α) decreased αLA stimulated GPR120 and GPR40 protein expression (Fig. 3). The decreased GLP-1 secretions could be due to receptor internalization. In addition, it has been shown that HIF-2α increased GPR40 in L-cells and potentiates fatty acid-induced GLP-1 secretion via extracellular signal-regulated kinase (ERK) [38]. In our study, under hypoxia, GPR120 and GPR40 were downregulated, which led to decreased GLP-1 secretion. Previously it has been shown that dual GPR120 and GPR40 agonist (DFL23916) delays receptor internalization and induce GLP-1 secretion in vitro and in vivo [6]*.* Internalization of receptors under hypoxia has been described in the brain before. Hypoxia-induced desensitization and internalization of adenosine A1 receptors in the rat hippocampus and internalization of Kappa (κ) opioid receptors [9].

## Conclusions

Hypoxia did not affect the basal GLP-1 secretion but decreased nutrient-stimulated GLP-1 secretion in enteroendocrine cells. In addition, hypoxia decreased the expression of long-chain fatty acid receptors GPR120 and GPR40. The decrease in nutrient-stimulated GLP-1 secretion could be due to downregulations of GPR120 and GPR40 receptors. Changes in the gut environment and inflammation might contribute to the hypoxia of the epithelial and L-cells.

## Data Availability

No datasets were generated or analysed during the current study.
